# Supplementary material for: Conformational Landscape of Cytochrome P450 Reductase Interactions
Source: Int J Mol Sci. 2021 Jan 20;22(3):1023. doi: 10.3390/ijms22031023 (PMC7864194; doi:10.3390/ijms22031023)
Supplement: Supplementary file 1 [file ijms-22-01023-s001.pdf]

Supporting Information: Conformational Landscape of Cytochrome P450 Reductase Interactions

Manuel Sellner, André Fischer, Charleen G. Don, and Martin Smieško*

Computational Pharmacy, Departement of Pharmaceutical Sciences, University of Basel, Klingelbergstrasse 61, 4056 Basel, Switzerland

E-mail: martin.smiesko@unibas.ch

Abstract

Oxidative reactions catalyzed by Cytochrome P450 enzymes (CYPs), which constitute the most relevant group of drug-metabolizing enzymes, are enabled by their redox partner Cytochrome P450 reductase (CPR). Both proteins are anchored to the membrane of the endoplasmic reticulum and the CPR undergoes a conformational change in order to interact with the respective CYP and transfer electrons. Here, we conducted over 22 microseconds of molecular dynamics (MD) simulations in combination with protein-protein docking to investigate the conformational changes necessary for the formation of the CPR–CYP complex. While some structural features of the CPR and the CPR–CYP2D6 complex we highlighted confirmed previous observations, our simulations revealed additional mechanisms for the conformational transition of the CPR. Unbiased simulations presented a movement of the whole protein relative to the membrane, potentially to facilitate interactions with its diverse set of redox partners. Further, we present a structural mechanism for the susceptibility of the CPR to different redox states based on the flip of a glycine residue disrupting the local interaction

network that maintains inter-domain proximity. Simulations of the CPR–CYP2D6 complex pointed toward an additional interaction surface of the FAD domain and the proximal side of CYP2D6. Altogether, this study provides novel structural insight into the mechanism of CPR–CYP interactions and underlying conformational changes improving our understanding of this complex machinery relevant for drug metabolism.

Results and Discussion

Open conformation of the CPR

Table S1: Production MD simualtions conducted in this study

**CPR state**[a]	**CYP**[b]	**Ligand**[c]	**Condition**[d]	**Duration**	**Replicas**
$NADP^+/FAD_{sq}/FMN_{sq}$	no	n/a	none	0.50 μs	1
FAD_{sq}/FMN_{sq}	no	n/a	none	1.44 μs	1
FAD_{hq}/FMN_{hq}	no	n/a	none	0.50 μs	1
FAD_{hq}/FMN_{sq}	no	n/a	none	0.20 μs	1
FAD_{sq}/FMN_{hq}	no	n/a	none	1.20 μs	1
FAD_{ox}/FMN_{sq}	no	n/a	none	1.00 μs	1
FAD_{ox}/FMN_{hq}	no	n/a	none	1.25 μs	3
FAD_{ox}/FMN_{hq}	no	n/a	500mM NaCl	0.48 μs	3
FAD_{ox}/FMN_{hq}	no	n/a	R243A	1.44 μs	3
FAD_{ox}/FMN_{hq}	no	n/a	p-Metadynamics	0.24 μs	1
FAD_{ox}/FMN_{hq}	no	n/a	wt-Metadynamics	0.24 μs	1
FAD_{ox}/FMN_{hq}	yes	none	none	0.30 μs	5
FAD_{ox}/FMN_{hq}	yes	APA	none	0.30 μs	5
FAD_{ox}/FMN_{hq}	yes	PMZ	none	0.30 μs	5
FAD_{ox}/FMN_{hq}	yes	TRA	none	0.30 μs	5
no	yes	none	none	0.30 μs	5

[a]Redox state of the CPR. [b]Presence of CYP2D6 in complex. [c]Ligand bound to CYP2D6 if complex was simulated. Either acetaminophen (APA), promethazine (PMZ), or tramadol (TRA). [d]Specific condition of the simulation. Metadynamics simulatons were conducted using either plain (p-Metadynamics) or well-tempered (wt-Metadynamics).

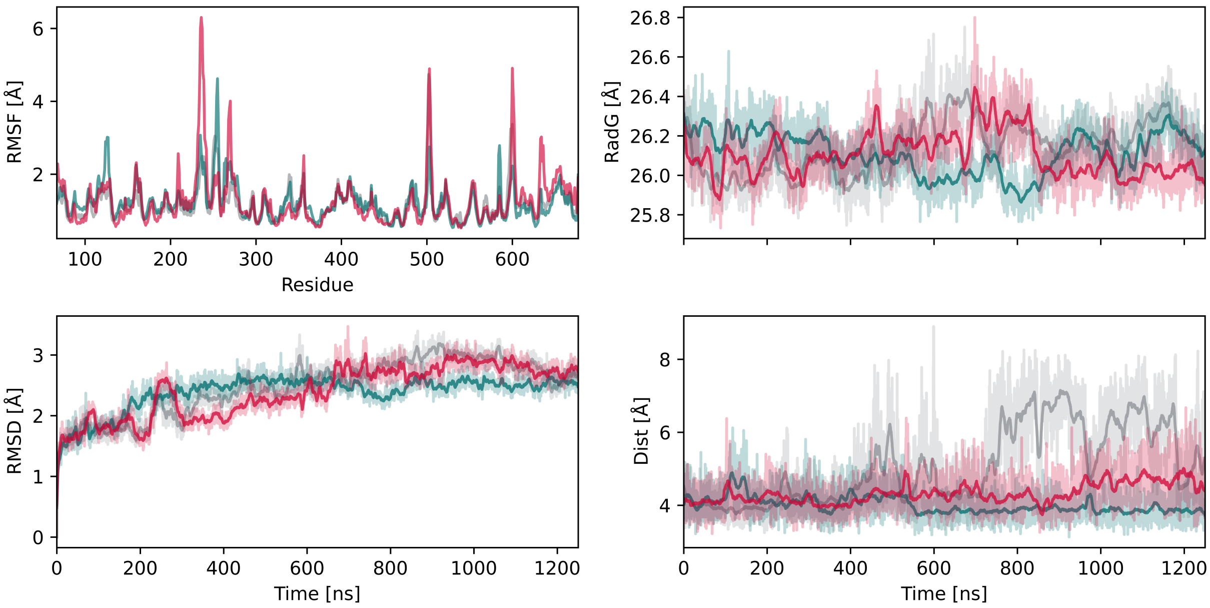

Figure S1: RMSF, RMSD, radius of gyration (denoted as RadG), as well as distance between FAD and FMN for the FAD_{ox}/FMN_{hq} system.

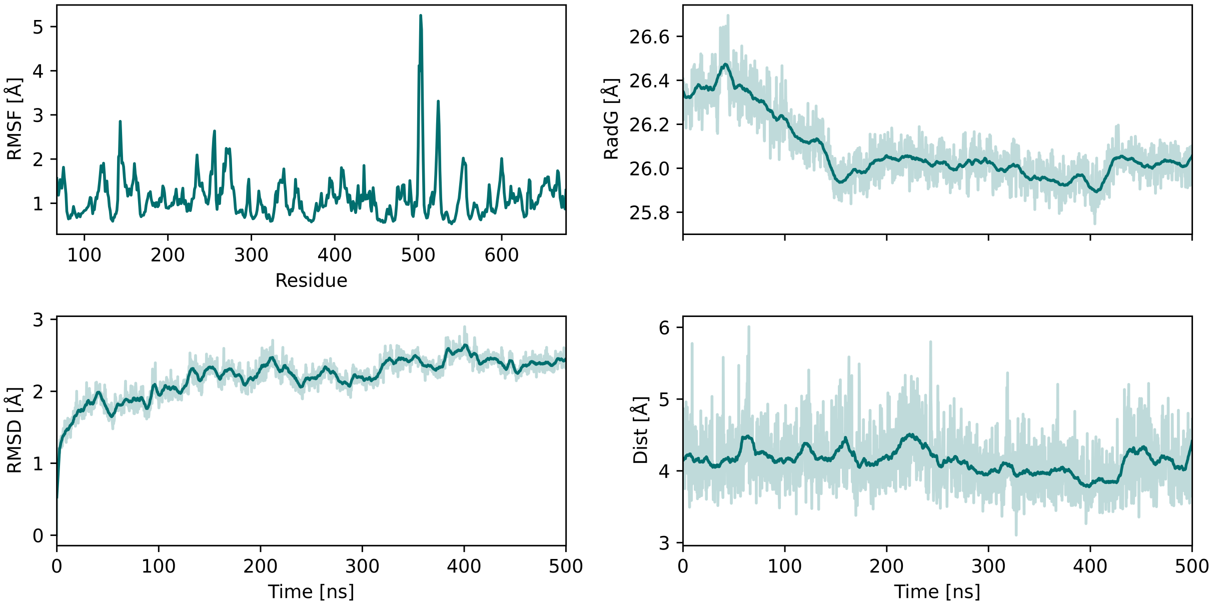

Figure S2: RMSF, RMSD, radius of gyration (denoted as RadG), as well as distance between FAD and FMN for the FAD_{hq}/FMN_{hq} system.

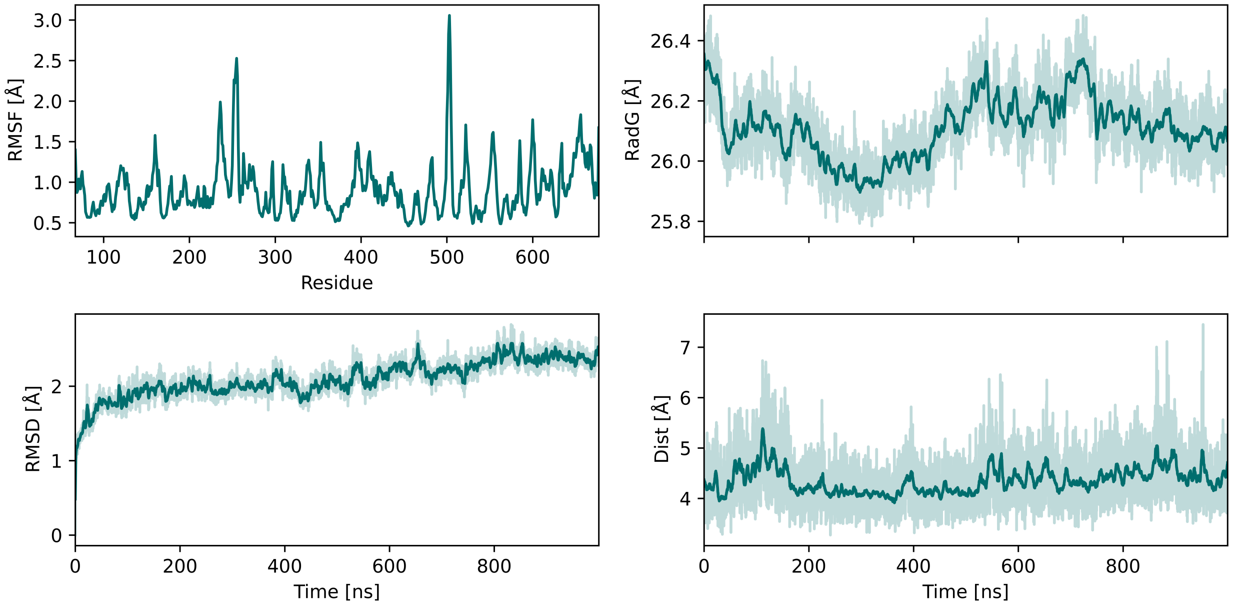

Figure S3: RMSF, RMSD, radius of gyration (denoted as RadG), as well as distance between FAD and FMN for the FAD_{ox}/FMN_{sq} system.

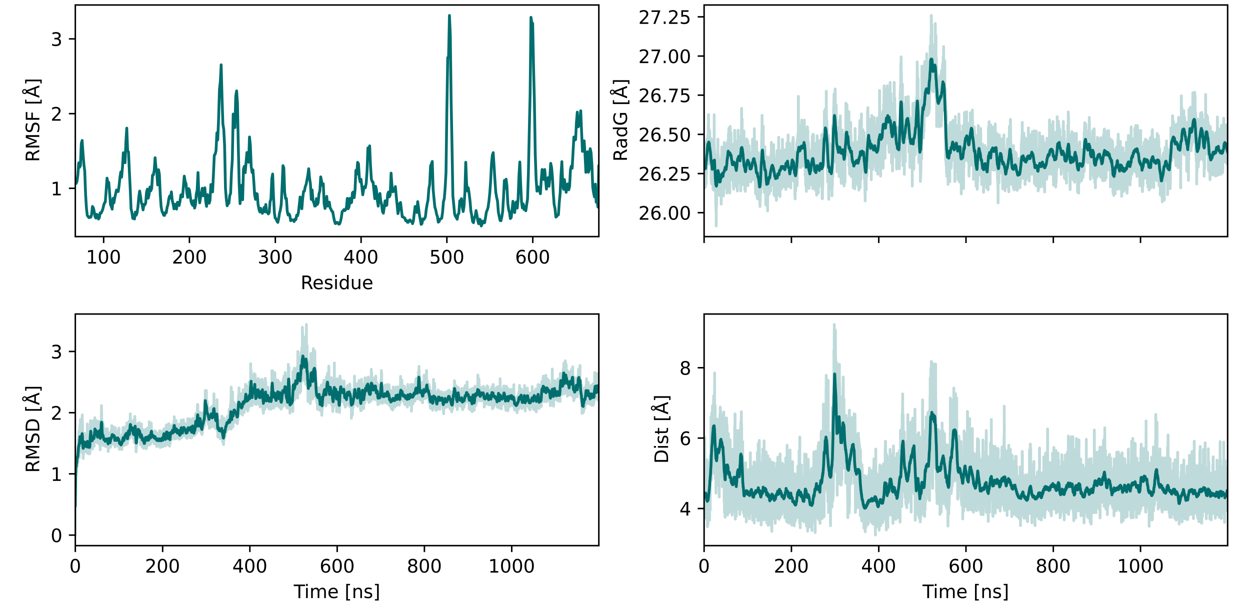

Figure S4: RMSF, RMSD, radius of gyration (denoted as RadG), as well as distance between FAD and FMN for the FAD_{sq}/FMN_{hq} system.

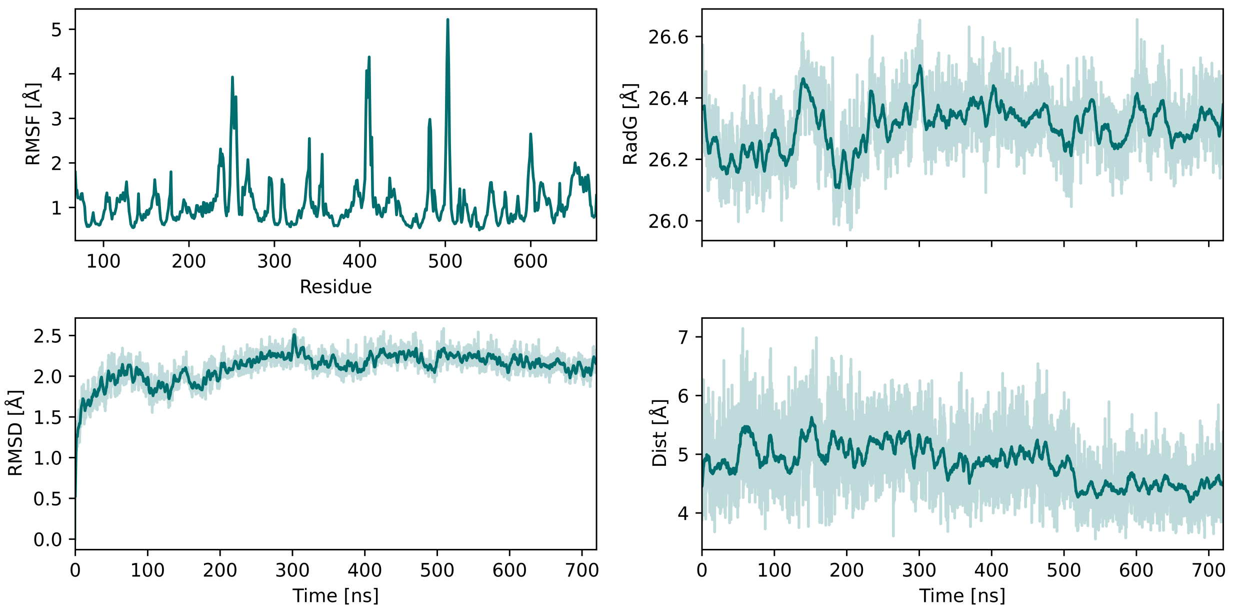

Figure S5: RMSF, RMSD, radius of gyration (denoted as RadG), as well as distance between FAD and FMN for the FAD_{sq}/FMN_{sq} system.

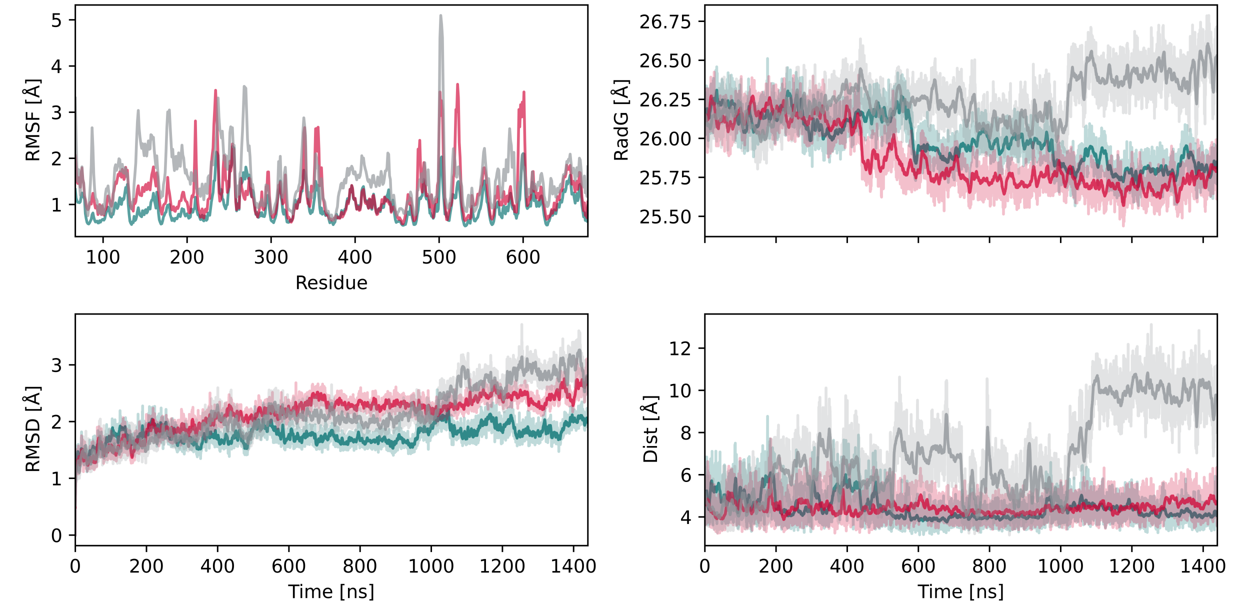

Figure S6: RMSF, RMSD, radius of gyration (denoted as RadG), as well as distance between FAD and FMN for the FAD_{ox}/FMN_{hq} R243A system.

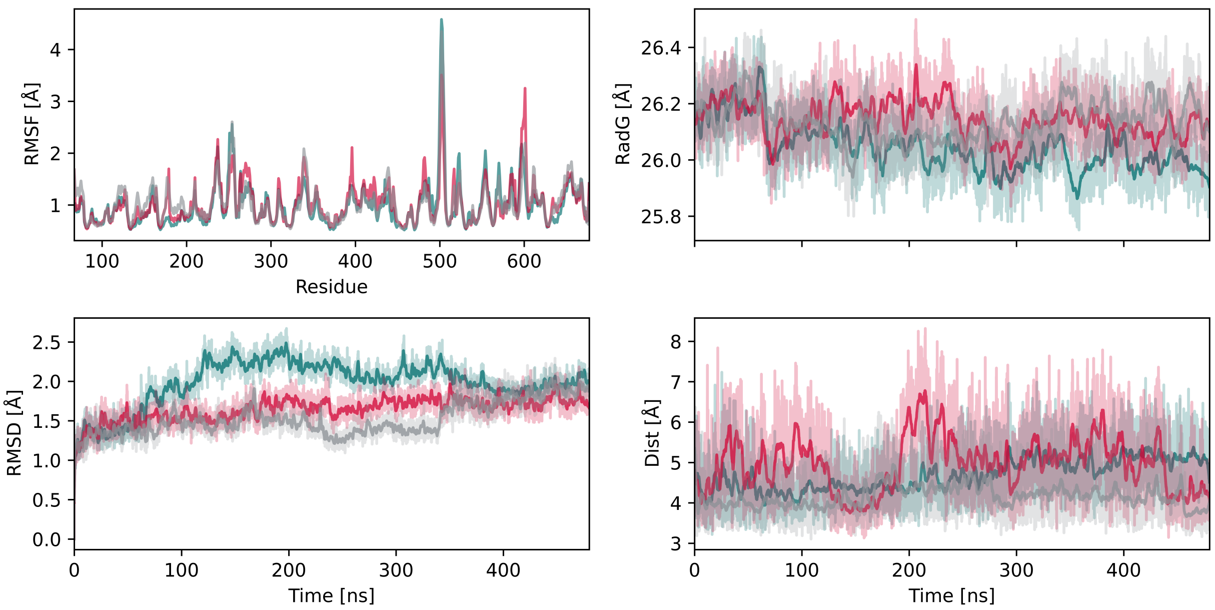

Figure S7: RMSF, RMSD, radius of gyration (denoted as RadG), as well as distance between FAD and FMN for the FAD_{ox}/FMN_{hq} NaCl system.

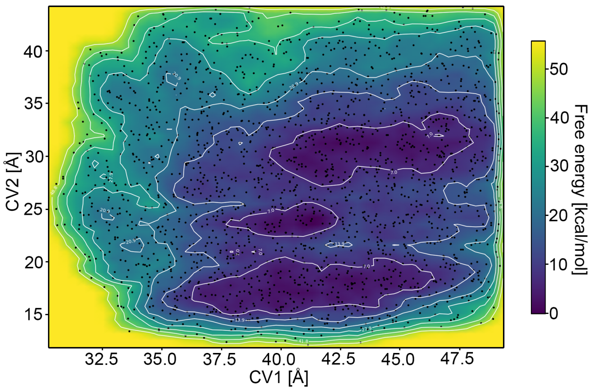

Figure S8: Free energy profile of plain metadynamics run.

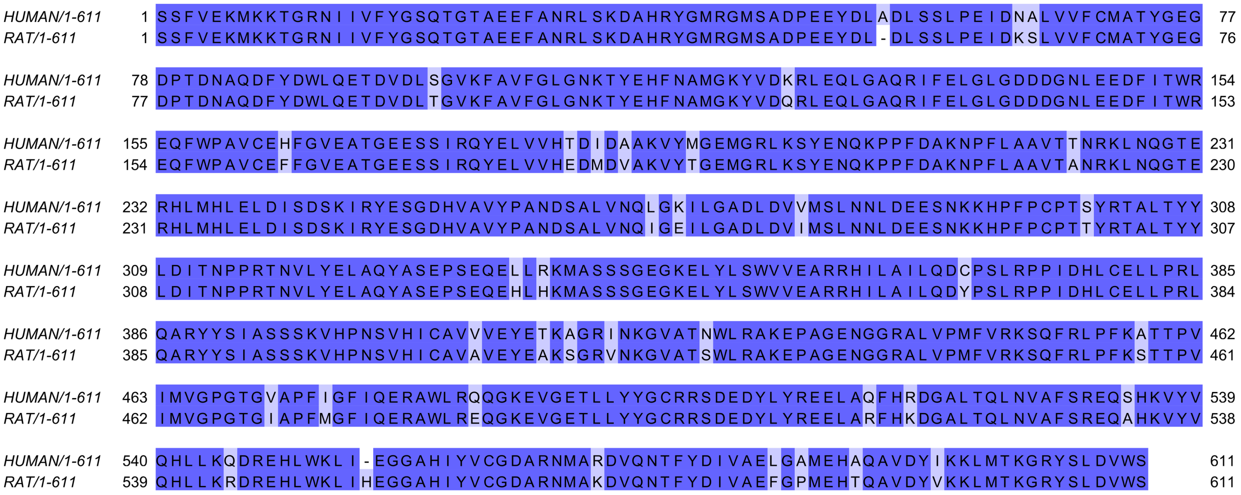

Figure S9: Sequence alignment of human and rat CPR.

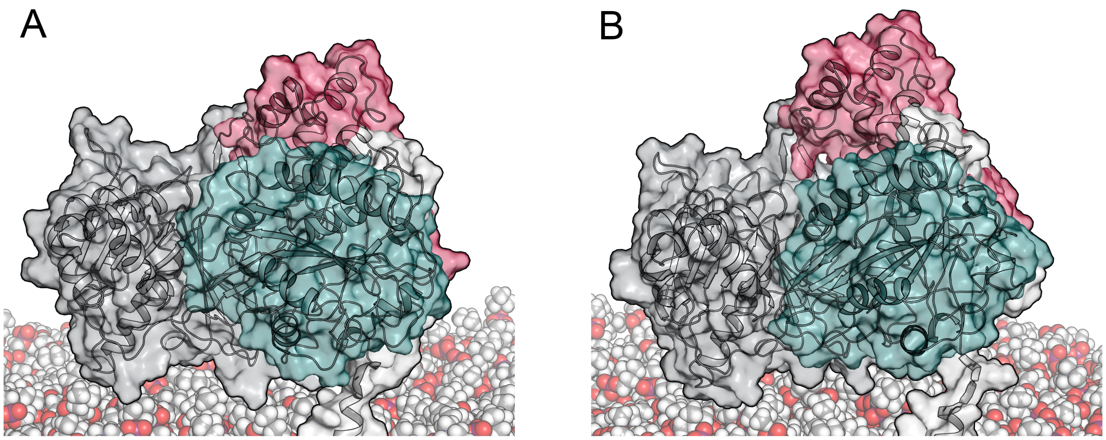

Figure S10: Closed conformations observed in metadynamics simulations. (A) Conformation with CV1 at 33.5 Å and CV2 at 27 Å, (B) Conformation with CV1 at 34 Å and CV2 at 35 Å.

Transitions between a sitting and upraised position

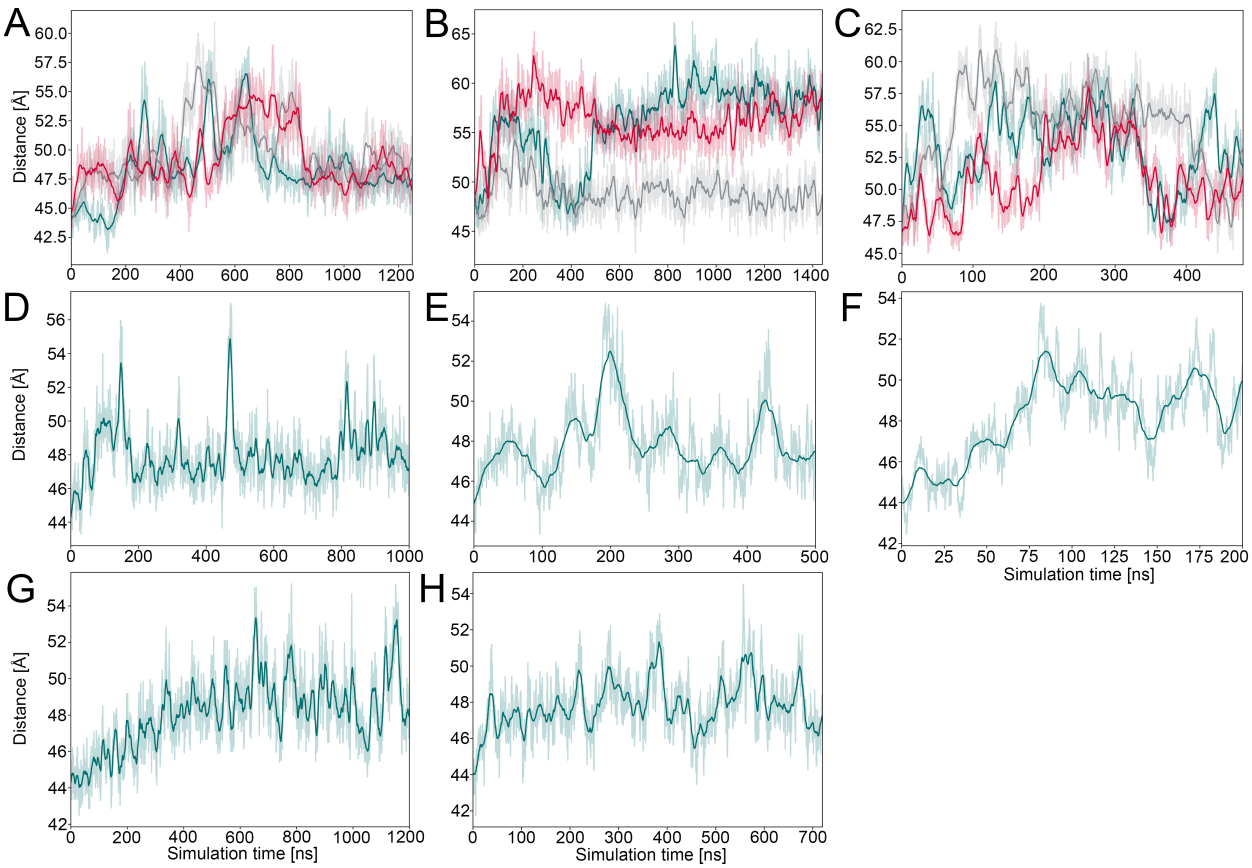

Figure S11: Distance between the centroid of the CPR and the centroid of the membrane in (A) FAD_{ox}/FMN_{hq}, (B) FAD_{ox}/FMN_{hq} with R243A mutation, (C) FAD_{ox}/FMN_{hq} with increased salt concentration, (D) FAD_{ox}/FMN_{sq}, (E) FAD_{hq}/FMN_{hq}, (F) FAD_{hq}/FMN_{sq}, (G) FAD_{sq}/FMN_{hq}, (H) FAD_{sq}/FMN_{sq} systems.

Glycine flip upon reduction of FMN

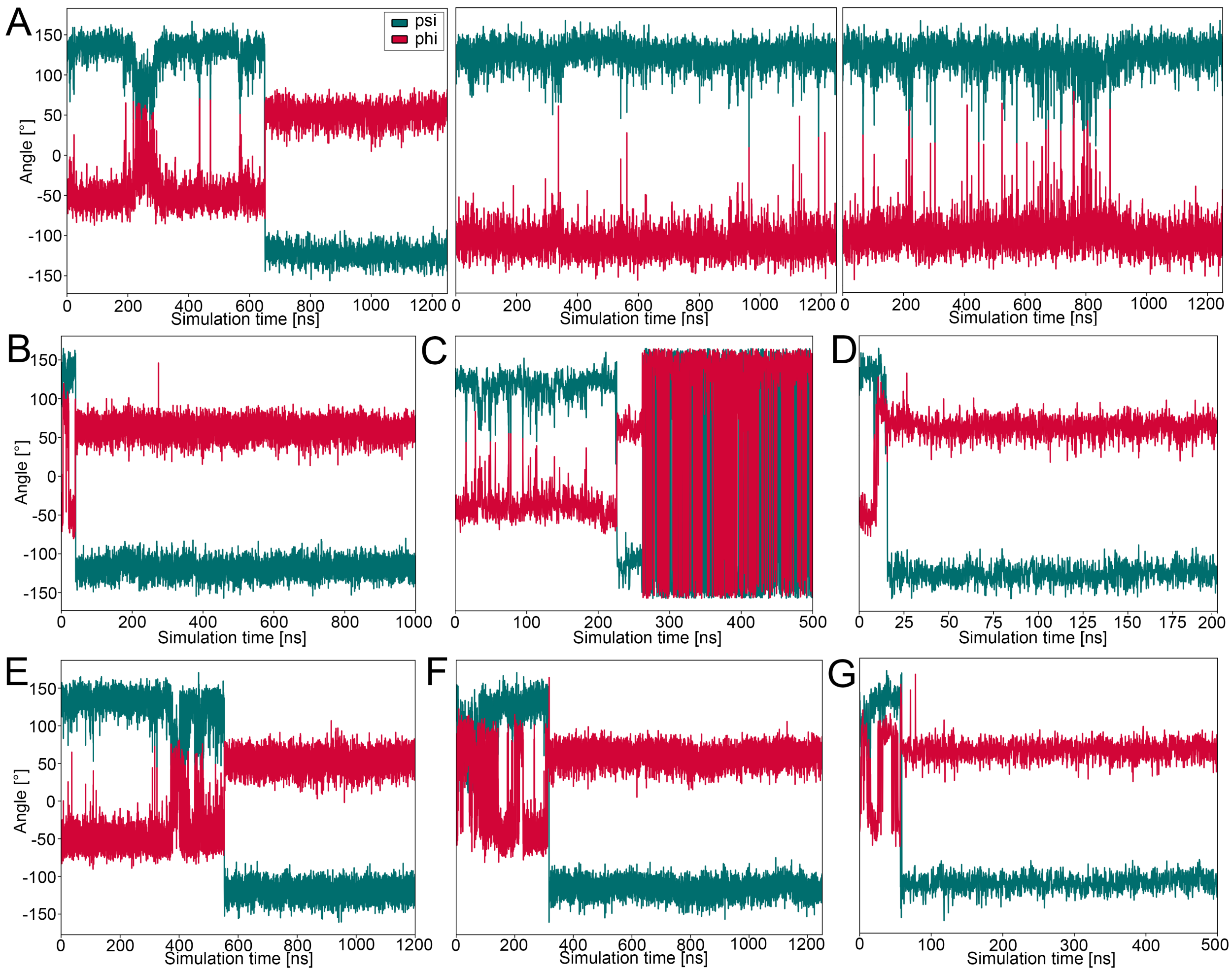

Figure S12: Backbone torsion angles of Gly141 to quantify its flip in simulations of (A) FAD_{ox}/FMN_{hq}, (B) FAD_{ox}/FMN_{sq}, (C) FAD_{hq}/FMN_{hq}, (D) FAD_{hq}/FMN_{sq}, (E) FAD_{sq}/FMN_{hq}, (F) FAD_{sq}/FMN_{sq}, (G) $FAD_{sq}/FMN_{sq}/NADP$ states.

Interactions between CYP2D6 and the CPR

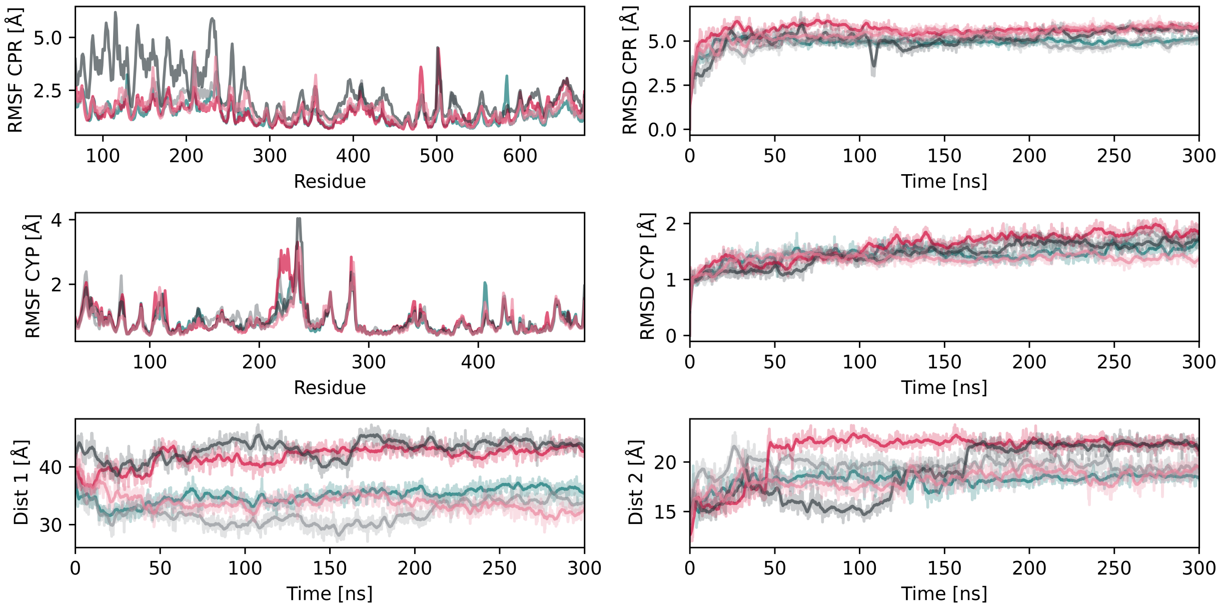

Figure S13: Metrics of the CPR–CYP2D6 acetaminophen bound complex simulations conducted with five replicas. The RMSD and RMSF of each protein is presented along the distance between FAD and FMN (denoted as Dist 1) and between the heme and FMN (denoted as Dist 2).

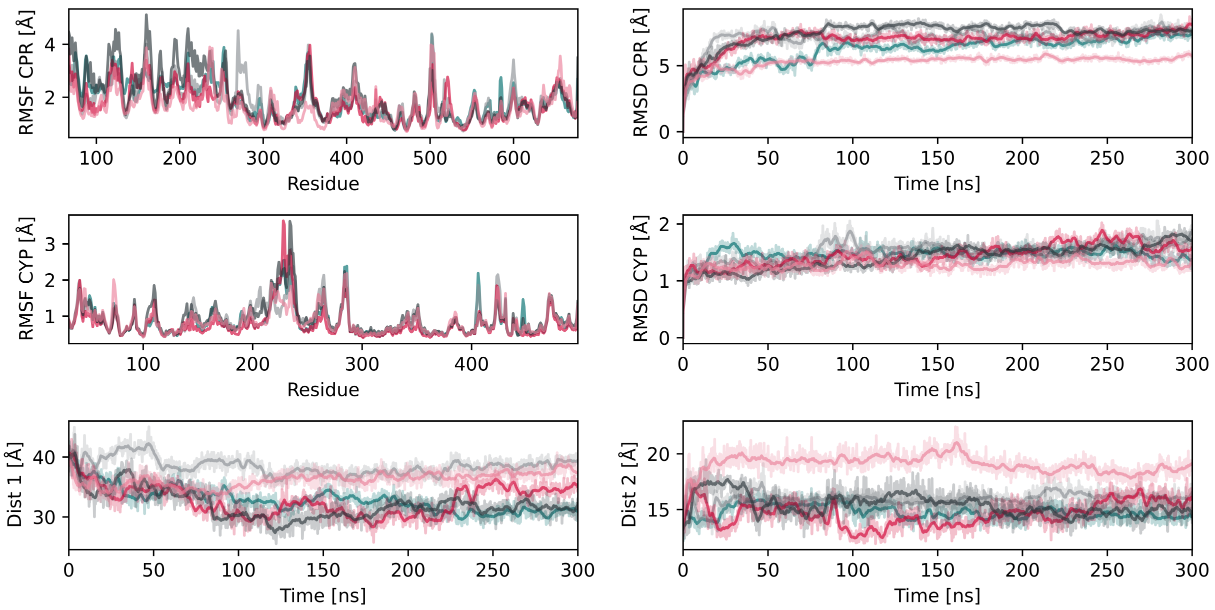

Figure S14: Metrics of the CPR–CYP2D6 promethazine bound complex simulations conducted with five replicas. The RMSD and RMSF of each protein is presented along the distance between FAD and FMN (denoted as Dist 1) and between the heme and FMN (denoted as Dist 2).

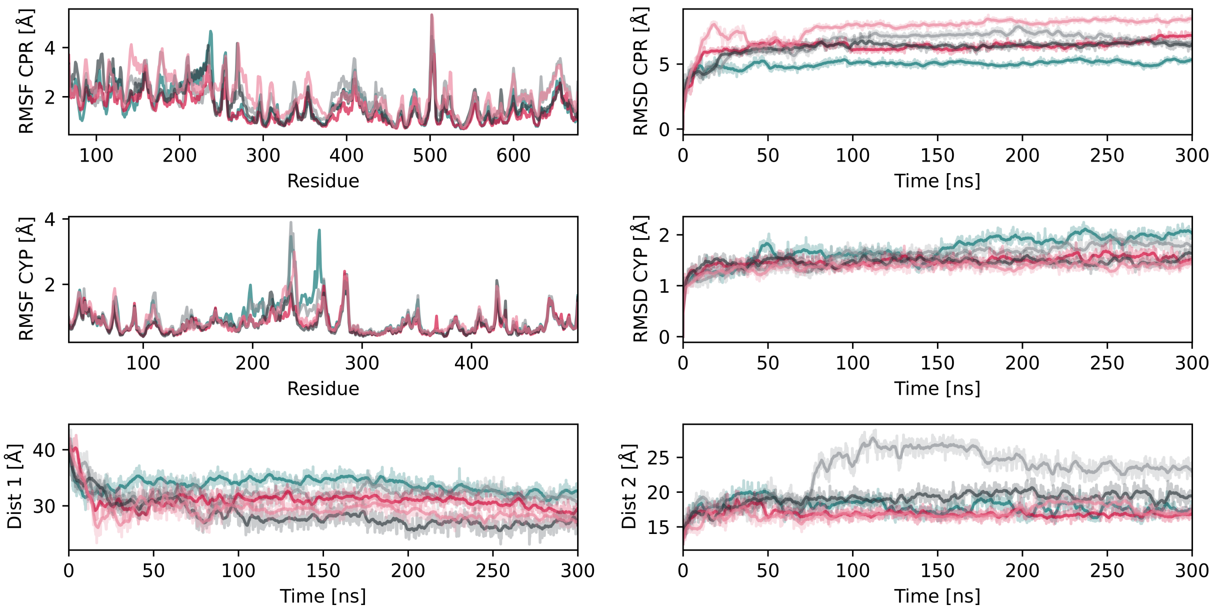

Figure S15: Metrics of the CPR–CYP2D6 tramadol bound complex simulations conducted with five replicas. The RMSD and RMSF of each protein is presented along the distance between FAD and FMN (denoted as Dist 1) and between the heme and FMN (denoted as Dist 2).

Table S2: Key interacting residues in the CPR and CYP2D6 forming intermolecular contacts in all simulations for at least 75% of the simulated time.

CYP2D6	CPR
Asp337	Lys523, Lys668, Arg670
Arg140	Glu142, Glu179
Arg440	Glu142, Asp144, Glu179

CPR binding affects tunnels in CYP2D6

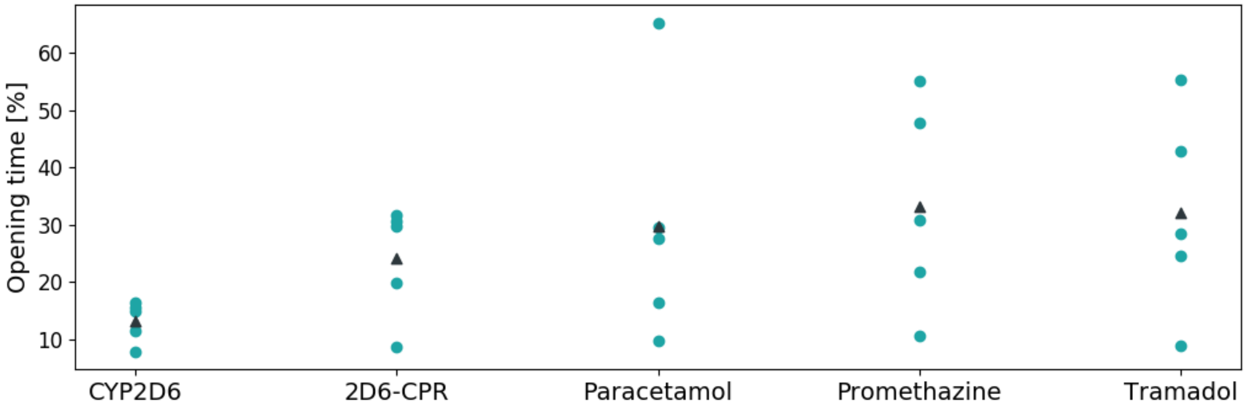

Figure S16: Relative opening times of tunnel 2f. CYP2D6 and 2D6-CPR represent the free CYP2D6 and the ligand-free ET complex, respectively. Paracetamol, promethazine, and tramadol represent the ET complex systems containing the according ligand. Data points are shown as dots while the corresponding mean values are displayed as triangles.

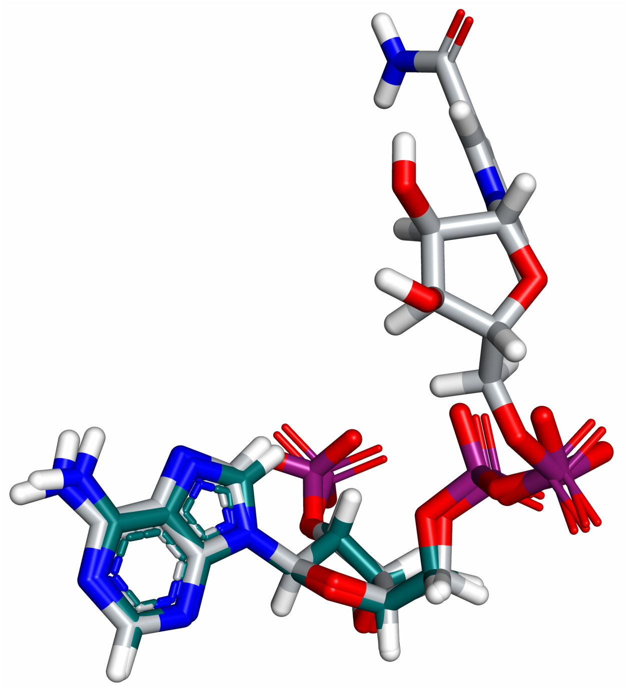

Figure S17: Alignment of NADPH. The partially resolved NADPH cofactor of the human CPR (pine green, PDB ID: 5FA6) is aligned with the fully solved NADPH cofactor of rat open CPR (grey, PDB ID: 3ES9)

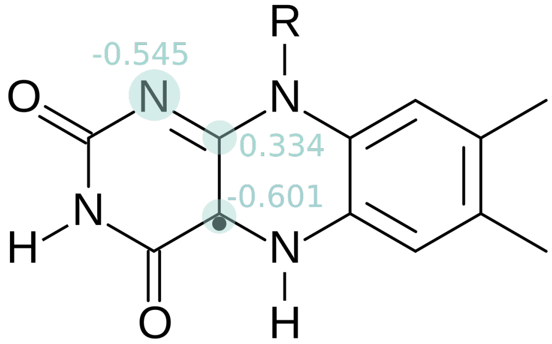

Figure S18: Adjusted partial charges for the flavin semiquinones.

Table S3: Active residues specified for protein–protein docking using Haddock.

**CYP2D6**	**FMN domain**	**FAD domain**
88, 129, 133, 140, 146, 326, 334, 336, 337, 346, 349, 429, 440	115, 116, 142, 144, 147, 208	514, 515, 519, 523, 664, 668, 670

Table S4: Clustering thresholds used for Caver 3.0.

	**CYP2D6**	**Complex 1[a]**	**Complex 2[b]**	**Complex 3[c]**	**Complex 4[d]**
Run 1	4.0	4.5	4.5	4.0	4.5
Run 2	4.5	4.5	4.0	4.5	4.5
Run 3	4.5	4.5	4.2	4.5	4.0
Run 4	4.5	4.5	5.0	4.5	4.5
Run 5	4.5	4.5	4.8	4.5	4.5

[a]2D6-CPR2. [b]2D6-CPR2 with paracetamol. [c]2D6-CPR2 with promethazine. [d]2D6-CPR2 with tramadol.
